# Effect of *Mucuna pruriens* Seed Extract Pretreatment on the Responses of Spontaneously Beating Rat Atria and Aortic Ring to *Naja sputatrix* (Javan Spitting Cobra) Venom

**DOI:** 10.1155/2012/486390

**Published:** 2011-07-11

**Authors:** Shin Yee Fung, Nget Hong Tan, Si Mui Sim, John C. Aguiyi

**Affiliations:** ^1^Department of Molecular Medicine, Faculty of Medicine, University of Malaya, 50603 Kuala Lumpur, Malaysia; ^2^Department of Pharmacology, Faculty of Medicine, University of Malaya, 50603 Kuala Lumpur, Malaysia; ^3^Department of Pharmacology and Clinical Pharmacy, Faculty of Pharmaceutical Sciences, University of Jos, P.M.B. 2084, Jos, Nigeria

## Abstract

*Mucuna pruriens* Linn. (velvet bean) has been used by native Nigerians as a prophylactic for snakebite. Rats pretreated with *M. pruriens* seed extract (MPE) have been shown to protect against the lethal and cardiovascular depressant effects of *Naja sputatrix* (Javan spitting cobra) venoms, and the protective effect involved immunological neutralization of the venom toxins. To investigate further the mechanism of the protective effect of MPE pretreatment against cobra venom toxicity, the actions of *Naja sputatrix* venom on spontaneously beating rat atria and aortic rings isolated from both MPE pretreated and untreated rats were studied. Our results showed that the MPE pretreatment conferred protection against cobra venom-induced depression of atrial contractility and atrial rate in the isolated atrial preparations, but it had no effect on the venom-induced contractile response of aortic ring preparation. These observations suggested that the protective effect of MPE pretreatment against cobra venom toxicity involves a direct protective action of MPE on the heart function, in addition to the known immunological neutralization mechanism, and that the protective effect does not involve action on blood vessel contraction. The results also suggest that *M. pruriens* seed may contain novel cardioprotective agent with potential therapeutic value.

## 1. Introduction

*Mucuna pruriens* Linn. (velvet bean) is found in Asia, America, and Africa. It is a popular medicinal plant in India [1] and contains protein (lectins, globulins, protease inhibitors), fat and fatty acids, water, fiber, and L-DOPA (or levodopa), among others. In certain regions of Nigeria, the beans have been prescribed by traditional practitioners as an oral prophylactic for snakebites [2]. The protective effect of the aqueous *M. pruriens* seed extract (MPE) has been demonstrated in mice against *Echis carinatus* and cobra venom [2, 3], and the protective effect was shown to involve an immunological neutralization mechanism. Indeed, preliminary studies have shown that anti-*M. pruriens* seed extract (anti-MPE) antibodies raised from rabbits were able to neutralize the lethalities of several Asiatic cobra venoms in mice [4]. Western blotting studies also showed that the anti-MPE IgG cross-reacted with purified neurotoxin and phospholipase A_2_ of *Naja sputatrix *(Javan spitting cobra) venom [5]. Further *in vivo *studies showed that rats pretreated with MPE were protected against the cardiovascular and respiratory depressant effects of *Calloselasma rhodostoma *(Malayan pit viper) and *N. sputatrix* venoms [6, 7]. Histological studies showed that one of the main features of the protective effect of MPE pretreatment in rats against the lethal effect of cobra venom was prevention of venom-induced histopathological changes in the heart [8]. This is of particular interest as clinical and experimental observations indicated that in cobra envenomation, cardiotoxicity may be a more prominent feature than neurotoxicity [9, 10]. 

While the major protective mechanism of MPE pretreatment appears to involve the neutralization of the venom toxins by anti-MPE antibodies elicited by the MPE pretreatment, the involvement of nonimmunological mechanism cannot be ruled out. In this study, we investigated the effect of MPE pretreatment on the responses of isolated heart atria and aortic rings to *N. sputatrix* venom as part of our attempt to understand the protective action of *Mucuna pruriens *seed extract on the cardiovascular system.

## 2. Materials and Methods

### 2.1. Plant Material and Seed Extract

*Mucuna pruriens* Linn. (family: *Fabaceae*, subfamily: *Papilionoideae*, genus: *Mucuna*, species: *pruriens*) seeds were collected from Rukuba area in Jos, Nigeria, with the aid of a traditional healer. They were authenticated by Professor S. W. H. Hussini of the Department of Botany, University of Jos. Voucher specimen Number A102 is deposited in the Pharmacy Herbarium of the University of Jos. The *M. pruriens* seed extract (MPE) was prepared according to Aguiyi et al. [2]. Briefly, dried *M. pruriens* seed meal (50 g) was soaked in distilled water (100 mL) for 24 h at 4°C with stirring. The extract was centrifuged at 10,000 g for 20 min, and the supernatant is termed *M. pruriens* seed extract (MPE). The extract was freeze-dried, and resuspended in normal saline prior to injection. MPE consists of both proteins and nonprotein components [3].

### 2.2. Venom, Drug Standards, and Chemicals

Lyophilized *N. sputatrix *(Javan spitting cobra, formerly known as *Naja naja sputatrix)* venom was purchased from Latoxan (Rosans, France), an established supplier of reliable venoms. The venom is a pooled sample from adult snakes and is from Indonesia. Carbachol (carbamylcholine chloride) and phenylephrine were purchased from Sigma Chemical Company (USA). All chemicals and reagents used in this study were of ACS grade. Stock solutions of all chemicals were prepared using ultrapure water. Dilutions of venom and drugs were made in normal saline.

### 2.3. Animals

Male Sprague Dawley rats (220–300 g) were used. All animals were handled according to guiding principles given by the Council for International Organization of Medical Sciences (CIOMS) on animal experimentation [11]. Animals were supplied by the Laboratory Animal Center of the Faculty of Medicine, University of Malaya, and the animal experimental protocol was approved by the Animal Care and Use Committee of the Faculty.

### 2.4. Determination of Median Lethal Dose (LD_50_)

The intravenous median lethal dose (LD_50_ (i.v.)) was determined by injection of various amounts of the venom into the caudal veins of rats (*n* = 5), and the lethal effect of the venom was observed for 24 h. The LD_50_ (i.v.) was then calculated according to the Spearman-Karber method [12].

### 2.5. Pretreatment of Rats

Rats were divided into two groups (*n* = 9 for each group). Pretreatment of rats was conducted according to the procedures of Guerranti et al. [3]. The treated group consisted of rats injected with MPE at a dose of 21 mg/kg (*i.p.*) at days 0, 7, and 14. The control (or untreated group) was given a similar volume of saline. After 21 days, the animals were killed, and isolated atria and aortic rings were prepared.

### 2.6. Preparation of Spontaneously Beating Rat Atria

Rats (pretreated and untreated) were stunned by a blow on the head and exsanguinated by cutting the carotid arteries. The paired rat atria were dissected out, cleaned of fatty tissues, and suspended in a 10-mL organ bath containing Krebs-Henseleit solution, maintained at 37°C, and aerated with 95% O_2_ and 5% CO_2_. The composition of Krebs-Henseleit solution was (mM): NaCl (118.4); NaHCO_3_ (25.0); MgSO_4_ (1.2); KCl (4.7); KH_2_PO_4_ (1.2); CaCl_2_ (2.5); glucose (11.1). Under resting tension of 1.0 g, the spontaneously beating atrial preparation was allowed to stabilize for at least 30 min, with change of the physiological salt solution every 15 min. Tension changes in the tissue were monitored via a force-displacement transducer (model FT03C) that was connected to MacLab data acquisition system (Version 3.5, Australia), and the rate of atrial beats were estimated from the recording.

### 2.7. The Action of *N. sputatrix* Venom on Isolated Spontaneously Beating Rat Atria from MPE-Pretreated and Untreated Rats

In two separate but parallel experiments, *N. sputatrix* venom was added at a concentration of 5, 50, and 250 *μ*g/mL cumulatively at 15 min interval to the tissues taken from untreated (as controls) and MPE-pretreated animals. The effects of the venom on the atrial contractility and rate were monitored at 5, 10, and 15 min for each concentration. At the end of the last 15 min, after exposure to 250 *μ*g venom/mL, the preparation was washed with physiological salt solution, and the atrial function was monitored for a further 15 min.

### 2.8. Preparation of Rat Aortic Rings

The thoracic aorta was gently excised, cleaned of surrounding fat and connective tissues, and then cut into 4 mm wide rings. Each aortic ring was then suspended in an organ bath between two parallel stainless steel hooks. One hook was fixed while the other was connected to a force-displacement transducer (model FT03C). The organ bath contained 10 mL of Krebs-Henseleit solution maintained at 37°C and aerated with 95% O_2_ and 5% CO_2_ throughout the experiment. The responses of the aortic ring were relayed to a MacLab data acquisition system (Version 3.5, Australia). A resting tension of 2 g was applied on the ring which was then allowed to equilibrate for 30 min before experimentation, with change of the physiological salt solution every 15 min. After equilibration, stimulation of the rings with 60 mM KCl was repeated until a stable contractile response was achieved.

### 2.9. Effect of *N. sputatrix* Venom on Aortic Rings of MPE-Pretreated and Untreated Rats

Endothelium intactness of aortic rings was checked by adding 2 *μ*M carbachol (carbamylcholine, CCh) to the preparation precontracted with 1 *μ*M phenylephrine (PE). Any preparation with <60% CCh-induced relaxation on a PE-precontracted aortic ring was discarded. Ten minutes after addition of carbachol, the aortic ring was washed at least three times with fresh Krebs-Henseleit solution and allowed to rest for 15 min. It was then exposed to *N. sputatrix* venom at 5, 50, and 250 *μ*g/mL, added cumulatively at 15 min interval, to observe the effects of the venom on the aortic rings prepared from untreated and MPE-pretreated animals. At the end of the experiment, the functional intactness of the endothelium of the aortic ring preparation was retested by repeating the effect of carbachol (2 *μ*M) on PE-induced contraction. Tissue responsiveness was also retested with 60 mM KCl.

### 2.10. Statistical Analysis

All biological data are presented as mean ± S.E.M. of *n* experiments. The effect of time or concentration was analyzed statistically using one-way repeated measures ANOVA, followed by Bonferroni/Dunnett T3 post-hoc test for multiple comparison (SPSS V14, SPSS Inc., Chicago, Ill, USA). The difference in the means between the untreated (control) and MPE-treated groups at any particular time or concentration was analyzed using Student's *t*-test. Statistical significance in the difference between means was indicated when *P* < 0.05.

## 3. Results

### 3.1. Determination of Median Lethal Dose (LD_50_)

The intravenous LD_50_ for *N. sputatrix* venom was determined to be 0.83 *μ*g/g in rats. The doses 5, 50, and 250 *μ*g/mL used in this study are therefore equivalent to approximately 0.3, 3.3, and 16.6 times of LD_50_, respectively, assuming that the total volume of blood in each young adult rat is approximately 20 mL.

### 3.2. Effect of *N. sputatrix* Venom on the Spontaneously Beating Atria Isolated from MPE-Pretreated and Untreated Rats

**Figure 1:**
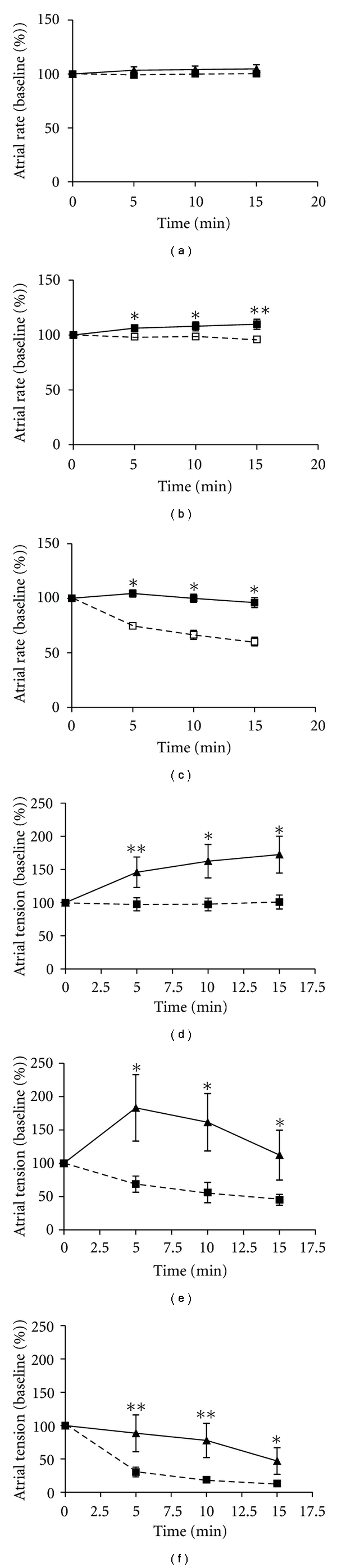
Effect of *Naja sputatrix* venom on the atrial rate (TOP, venom concentration (a) = 5 *μ*g/mL, (b) = 50 *μ*g/mL, and (c) = 250 *μ*g/mL) and atrial contractility (BOTTOM, venom concentration (d) = 5 *μ*g/mL, (e) = 50 *μ*g/mL, and (f) = 250 *μ*g/mL) of isolated atria from untreated (dotted line) and MPE-pretreated rats (solid line). The isolated atrial preparations were suspended in a 10 mL organ bath containing Krebs-Henseleit solution maintained at 37°C and aerated with 95% O_2_ and 5% CO_2_. The effect of the venom on the atrial rate and contractility was monitored for 15 min for each concentration. Values represent mean ± S.E.M. (*n* = 9). *P < 0.05, **P < 0.01, ***P < 0.001 compared to the untreated animals at the corresponding time using unpaired Student's *t*-test. One way repeated measures ANOVA followed by Bonferroni/Dunnett T3 post-hoc test were used to determine the effect of time on the atrial rate and contractility responses for each venom concentration. The effect of time was only significant at 250 *μ*g/mL in the untreated group, *P* < 0.001.

*N. sputatrix* venom caused both time-dependent and venom concentration-dependent changes on the atrial contractility and rate (Figures 1(a)–1(c): results expressed as % baseline g tension and % baseline beats per minute, resp.). The reduction of atrial rate was minimal at both 5 and 50 *μ*g/mL and became marked only at 250 *μ*g/mL (*P* < 0.001). At this highest venom concentration tested, the atrial rate was reduced by 25, 34, and 40% from baseline, for the three incubation time points of 5, 10, and 15 min, respectively. These reductions in atrial rate were completely prevented in the atria isolated from MPE-pretreated rats (Figure 1(c), solid lines). At 5 *μ*g/mL, the venom induced minimum change in the atrial rate of untreated rats. There was no difference between the atrial rates of the MPE-pretreated and untreated groups at all three monitored time points (Figure 1(a), *P* > 0.05). At 50 *μ*g/mL, there was a decreasing trend in the atrial rate response to the venom in the untreated group whereas the MPE-pretreated group actually showed an increasing trend in the atrial rate response to the same dose of venom (Figure 1(b)). The difference between the means of the two groups was small but significant at each of the three incubation time points (*P* < 0.05).

The effect of the venom on the contractility of the isolated atria was more marked than that on the heart rate (Figures 1(d)–1(f)). At a concentration of 250 *μ*g/mL, the venom drastically reduced the contractility of the untreated (control) rat atria by 70, 81, and 87% at 5, 10, and 15 min incubation time, respectively (*P* < 0.001). In contrast, the same venom concentration did not significantly reduce the contractility of the atria from MPE-pretreated rats although there was a downward trend over the 15 min incubation period (Figure 1(f)). At the two lower concentrations studied, the venom reduced progressively the contractility of atria from the untreated group whereas the venom showed a tendency to increase the contractility of atria from MPE-pretreated rats by 13–83% above the baseline (Figures 1(d) and 1(e)). 

The depressed atrial contractility and rate of both untreated and MPE-pretreated rats did not return to their respective baseline values even after repeated washings of the preparation.

### 3.3. Effect of *N. sputatrix* Venom on Aortic Rings of MPE-Pretreated and Untreated Rats

**Figure 2:**
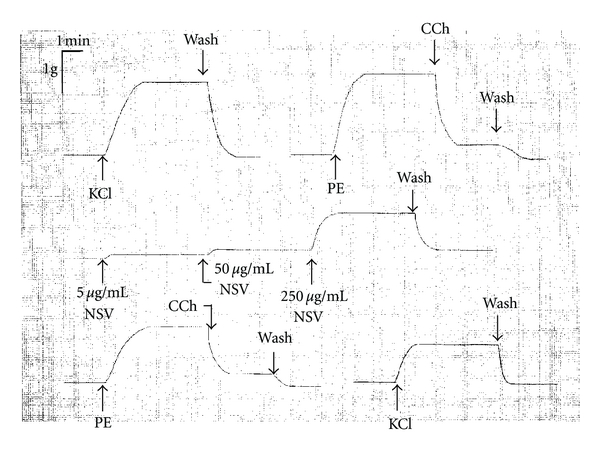
Representative tracing showing the effects of different concentrations of *Naja sputatrix* venom (NSV) on the endothelium-intact aortic ring isolated from untreated rat. Cumulative additions of the *N. sputatrix* crude venom, at final bath concentrations of 5, 50, and 250 *μ*g/mL, induced a contractile effect on the aortic ring preparation. Subsequent to testing the venom, the preparation was repeatedly washed with fresh Krebs-Henseleit solution. Potassium chloride (KCl), phenylephrine-(PE-) and carbamylcholine chloride (CCh) were given before and after the venom dose to check for the tissue viability and endothelium integrity.

To assess the tissue viability and functional integrity of the endothelium, carbachol (CCh, 2 *μ*M) was added to the endothelium-intact aortic ring at the peak of phenylephrine (PE)-induced contraction. A near complete relaxation (80.9 ± 4.7%, *n* = 18) of the ring indicated that the endothelium preparation was functionally intact (Figure 2, representative tracing). In a preliminary study, this venom (10 and 100 *μ*g/mL) was found to produce a similar dose-dependent contraction when given at resting tension and when given to a PE-precontracted aortic ring. No relaxation response was observed. Therefore, all subsequent studies involved giving cobra venom at resting tension only. In the present study, the venom induced a concentration-dependent contractile response on the aortic ring from the untreated rats (Figure 2 and Table 1). These contractile responses induced by venom given at resting tension, however, were not significantly altered by MPE-pretreatment at all the three venom concentrations tested (*P* > 0.05, Table 1).

## 4. Discussion

Earlier studies showed that the protective effect of *M. pruriens *seed extract (MPE) pretreatment against the lethal activities of *E. carinatus* and *N. sputatrix* venoms involved an immunological mechanism. The pretreatment elicited formation of antibodies in the mice or rats that were capable of neutralizing certain snake venom toxins [3, 4]. Fung et al. [6] reported that rats pretreated with the MPE were protected against the cardiovascular depressant effects of *N. sputatrix* venoms, including depression in blood pressure and heart rate. This present paper reports our effort to further investigate the protective mechanism(s) of the MPE pretreatment. Specifically, we aim to answer two questions: Firstly, is immunological neutralization of venom toxins the only mechanism by which MPE pretreatment protect against cobra venom? Secondly, does the protective action of MPE pretreatment against the venom-induced cardiovascular effect (in particular the hypotensive effect) involve the heart alone, or does it involve the blood vessels as well? To answer these two questions, we investigated the effects of *N. sputatrix* venom on isolated spontaneously beating atria and aortic rings, taken from MPE-pretreated and untreated rats. 

Our studies using isolated atrial preparations showed that the MPE pretreatment conferred protection against cobra venom-induced depression of heart rate and contractility, at both 50 *μ*g/mL (equivalent to 3.3 LD_50_) and 250 *μ*g/mL (equivalent to 16.6 LD_50_). At 5 *μ*g/mL (equivalent to 0.3 LD_50_), the amount of venom used was too low to cause any observable adverse effects on atrial contractility and rate from untreated rats. These results showed that the protection against venom-induced hypotensive effect in the MPE-pretreated rats reported in the anesthetized animal study [7] involves protection against venom-induced harmful effects to the heart. This protective action of MPE-pretreatment, however, was not sufficient to prevent venom-induced damages to the heart at very high venom concentration, as repeated washings after the 250 *μ*g/mL treatment failed to restore the atrial contractility and rate to the baseline, indicating that the venom has already caused permanent damages to the heart. This is consistent with the earlier report that lethal dose of *N. sputatrix* venom can lead to histopathological changes in cardiac muscle [8].

In addition, the present results also support our hypothesis that immunological neutralization is not the only mechanism involved in the protective effect of MPE pretreatment against cobra venom, as the experiments reported here were *in vitro* experiments using isolated atrial preparations, and there was no serum (and hence no anti-MPE antibodies) present in the tissue bath. The mechanism of cobra venom-induced cardiac toxicities is still not fully understood, even though it certainly involves the venom cardiotoxin and phospholipases A_2_ [13, 14]. It is important to note that the present studies involved the action of *N. sputatrix* venom on isolated atrial preparations isolated from animals sacrificed 7 days after the last MPE treatment. The effects of seed constituents such as L-DOPA (which can produce dopamine with positive chronotropic and positive inotropic effects on the heart) and other small molecule, water-soluble xenobiotic compounds on the animal are relatively short-term and will not be expected to have an effect 7 days after administration. 

It would appear that the MPE pretreatment has a direct action on rat atria, which may induce gene expression changes to enhance the heart's resistance to the cardiotoxic effect of cobra venom toxins. Interestingly, the contractility of the atrial preparations from MPE-pretreated rat increased transiently on exposure to low doses of the cobra venom (5 and 50 *μ*g/mL). The mechanism of this unexpected increase in atrial contractility is unclear, but it is probably related to some adaptive response of the atria (from the pretreated rats) to venom exposure. The phenomenon supports the suggestion that MPE pretreatment has a direct action on rat heart, rendering it more resistant to venom-induced cardiotoxicity. 

The results from the *in vitro *aortic ring isolated from untreated rats showed that *N. sputatrix* venom caused contraction of the aortic ring in a concentration-dependent manner, which may be due to interactions between venom toxins and postsynaptic receptors in the blood vessel. Alternatively, the venom toxins could act directly on the musculature to depolarize the membrane, leading to an increase in intracellular Ca^2+^ level and ultimately a contractile response. 

Aortic rings isolated from MPE-pretreated rats demonstrated the same concentration-dependent contraction showing that MPE pretreatment does not protect against the vasoactive action of cobra venom.

In conclusion, our results suggest that the protective effect of MPE pretreatment against cobra venom involves a direct action of MPE on the heart, and, as such, immunological neutralization is not the only mechanism of protective effect of the MPE pretreatment. Furthermore, the protective action of MPE pretreatment against cobra venom-induced cardiovascular depressant effect is due to its action on the heart and does not involve the blood vessel. Future molecular and gene expression studies are being undertaken to elucidate further the mechanism of the cardioprotective action of the MPE pretreatment and to identify the MPE constituent(s) responsible for the action, which could lead to discovery of novel cardioprotective agent(s).

## Figures and Tables

**Table 1:** Effects of *Naja sputatrix* venom on the contraction of aortic rings isolated from MPE-pretreated and untreated rats.

Venom concentration (*μ*g/mL)	Aortic ring contraction	
	(% phenylephrine contraction)	
	Untreated	MPE-pretreated
5	3.5 ± 1.6	5.3 ± 2.0
50	16.5 ± 2.9^##^	14.6 ± 2.7^#^
250	112.5 ± 14.9^###^	115.7 ± 15.4^###^

Values represent mean ± S.E.M. (*n* = 9). The effect of concentration was analysed using one-way repeated measures ANOVA followed by Benferroni/Dunnett T3 post-hoc test. ^#^ *P* < 0.05, ^##^ *P* < 0.01, ^###^ *P* < 0.001 compared to 5 *μ*g/mL. The difference in the means between the untreated and the MPE-pretreated groups was not significant at all three venom concentrations. *P* > 0.05, unpaired Student's *t*-test.
